# Evaluation of Anal Cancer Screening Among 18-to-34-Year-Old Men Who Have Sex With Men Living With HIV Over a 10-Year Period

**DOI:** 10.1093/ofid/ofag418

**Published:** 2026-07-10

**Authors:** Serina S Applebaum, Elizabeth Chan, Michael Virata, Ritche Hao, Lydia Barakat, Amit C Achhra

**Affiliations:** Section of Infectious Diseases, Department of Medicine, Yale School of Medicine, New Haven, Connecticut, USA; Section of Infectious Diseases, Department of Medicine, Yale School of Medicine, New Haven, Connecticut, USA; Atrius Health, Medford, Massachusetts, USA; Section of Infectious Diseases, Department of Medicine, Yale School of Medicine, New Haven, Connecticut, USA; Section of Infectious Diseases, Department of Medicine, Yale School of Medicine, New Haven, Connecticut, USA; Section of Infectious Diseases, Department of Medicine, Yale School of Medicine, New Haven, Connecticut, USA; Section of Infectious Diseases, Department of Medicine, Yale School of Medicine, New Haven, Connecticut, USA

**Keywords:** anus neoplasms, early detection of cancer, HIV infections, papillomavirus infections, sexual and gender minorities

## Abstract

**Background:**

Guidelines now support anal squamous cell carcinoma (ASCC) screening in men who have sex with men (MSM) living with HIV (LWH) aged ≥35 years. There is limited data on screening in MSM LWH <35 years.

**Setting:**

Two academic HIV clinics in the Northeastern US.

**Methods:**

We retrospectively evaluated all anal cytology and high-resolution anoscopy (HRA; referral criteria: atypical cells of undetermined significance [ASC-US] or worse cytology) performed for MSM LWH between ages 18–34 from 2013 to 2023. We evaluated risk factors for abnormal cytology or histology, and prevalence of high-grade dysplasia (HSIL) and ASCC.

**Results:**

Of 216 eligible MSM LWH, 111 (51%) were screened for ASCC at least once. Screened individuals had longer follow-up (median 4.0 vs 1.6 years) but were otherwise similar to unscreened individuals. Among 246 cytology tests, 19% were unsatisfactory and 31% abnormal, of which 1.2% reported HSIL. Of 23 HRAs following abnormal cytology, 10 (43%) revealed HSIL. Testing in response to symptoms was associated with abnormal results (OR 8.54; 95% CI 2.55–28.67). Human papillomavirus vaccination trended toward protection against abnormal results. We identified two cases of local ASCC, both in individuals with symptomatic condyloma and severe immunosuppression.

**Conclusions:**

Over 10 years, ASCC screening in MSM LWH <35 years was variable. Anal cytology had high unsatisfactory rates and poor correlation with histology. Despite high dysplasia prevalence among those undergoing HRA, ASCC was rare and diagnosed early from symptoms. Findings support excluding younger (≤34 years) MSM LWH from asymptomatic screening, and support symptom-driven testing to diagnose anal cancer early.

## Teaser

Among MSM with HIV aged 18–34, cytology for anal cancer screening had high unsatisfactory rates and poor correlation with histology. HSILs were frequent, but anal cancer was rare and detected early from symptoms. Findings support current guidelines in excluding this group from asymptomatic screening.

Anal squamous cell carcinoma (ASCC) is a rare but significant health concern, with a disproportionately high burden among men who have sex with men (MSM) living with HIV-1 (LWH). MSM LWH have an estimated anal cancer incidence of 85 per 100 000 person-years, comparable to cervical cancer rates prior to widespread cervical cytology screening [1, 2]. Screening for ASCC typically involves cytologic evaluation (via anal Papanicolaou test) to identify precancerous changes, followed by high-resolution anoscopy (HRA) for abnormal findings. Digital rectal examination (DRE) and high-risk HPV (hrHPV) testing are also recommended and used in clinical practice. Recent guidelines from the International Anal Neoplasia Society (IANS) and US Department of Health and Human Services (DHHS) recommend annual anal cytology for MSM LWH aged ≥35 years in combination with DRE and/or hrHPV testing, to detect and treat high-grade squamous intraepithelial lesions (HSIL) before progression to cancer [3, 4]. This age threshold is based on the landmark Anal Cancer–HSIL Outcomes Research (ANCHOR) trial demonstrating reduced ASCC incidence with treatment of HSIL by HRA in people with HIV aged >35 years, in conjunction with epidemiological evidence suggesting increased risk of HSIL and anal cancer with age, particularly after age 60 [1, 5–7].

The age cutoff for cancer screening has decreased over time for other malignancies such as breast, colorectal, and lung cancer [8, 9]. Some authors have proposed consideration of earlier ASCC screening in MSM LWH, to more closely align with the recommended cervical cancer screening age for women at 21 years old [2, 10, 11]. This is likely driven by findings of high prevalence of HSIL even in younger MSM cohorts. Some country guidelines (eg, France) and some institutes do offer screening to younger MSM LWH [12–14]. Anecdotally, queries about earlier screening often arise from patient communities and providers as well. However, empiric evaluation supporting routine screening of MSM LWH under age 35 is limited [6].

## METHODS

Prior to the release of recent national guidelines, many centers, including those in this study, routinely offered screening to all adult MSM LWH, including those younger than the current guideline cutoffs [15]. We retrospectively evaluated the prevalence and outcomes of anal cancer screening with anal cytology among MSM LWH aged 18–34. Specifically, we aimed to describe the uptake of screening, prevalence of cytologic abnormalities and histologic HSIL, and assess factors associated with abnormal screening results in this younger cohort. To contextualize these findings, we also report documented ASCC cases in the MSM LWH 18–34 age group from Yale's tumor registry, under which both of the study clinics as well as other regional Yale system clinics fall.

### Setting

We conducted this study at two infectious disease clinics affiliated with a large tertiary-care urban academic health system serving a socio-demographically and economically diverse population in the greater New Haven, CT area. These clinics operated independently for most of the study period but merged into a single clinic in late 2023. Together, they provide care to approximately 1500 people LWH. Approximately 40% of the clinic population LWH are MSM and 20% are aged 18–34. The Yale Institutional Review Board approved the study and waived the requirement for informed consent. Screening practices varied by provider due to the absence of national guidelines during the study period and may have resembled contemporary New York State Department of Health guidelines [16]. HrHPV was not routinely tested during the study period and therefore varied by provider preference. After recent anal cancer screening guidelines were published, co-testing for hrHPV was increasingly performed as part of the guideline congruent screening algorithm [4]. Cytology was typically collected using the EndoCervex-Brush (Rovers Medical Devices, Oss, The Netherlands) with BD SurePath (Beckton Dickinson, Franklin Lakes, NJ) as the collection vial. Results were categorized according to the Bethesda System as unsatisfactory, negative, atypical squamous cells of undetermined significance (ASC-US), low-grade squamous intraepithelial lesion (LSIL), atypical squamous cells, cannot exclude HSIL (ASC-H), or HSIL [17]. Interpretation came from Yale New Haven Health cyto-pathology. Unsatisfactory cytology results were generally followed by repeat cytology. Though screening and referral practices varied by provider, clinicians at both sites generally used a low-threshold approach to HRA referral, including anyone with abnormal cytology, defined as ASC-US, LSIL, ASC-H, or HSIL. Per colorectal section procedure, a colorectal surgeon performed HRAs in the operating room, as clinic based HRA was not yet available. We recorded biopsy reports using Lower Anogenital Squamous Terminology (LAST) as negative, LSIL, or HSIL. HSILs were generally treated with ablation or topical therapy, and follow-up with periodic HRA or cytology at the clinical discretion of the surgical team.

### Patient Cohort and Data Collection

We queried the electronic health record (EHR) from 2013 to 2023 to identify individuals with HIV, assigned male at birth, who self-identified as gay or bisexual, and received care at the infectious disease clinic between the ages of 18 and 34. We excluded encounters occurring at or after age 35. We recorded characteristics for the entire cohort including age, race and ethnicity, total follow-up duration (defined as time in years between first and last visits to the clinic), insurance status, CD4 T-cell count, HIV viral load, HPV vaccination status, and smoking history. We obtained CD4 T-cell count and HIV viral load as the most recently recorded value and categorized to reflect immune function and virologic suppression, consistent with clinically relevant thresholds [3]. We recorded all anal cytology and HRA biopsy results between January 2013 and August 2023. We also queried the EHR for incident cases of ASCC and cross-referenced the cohort for diagnoses in the Yale Cancer Center Tumor Registry, which contains all reportable cancer patients diagnosed or treated at Smilow Cancer Hospital at Yale-New Haven and Yale Cancer Center, with a larger catchment area of system wide clinics (including the two clinics in this study). The Tumor Registry contained 344 instances of ASCC between 2013 and 2024, which we further filtered to our study inclusion criteria.

We determined from chart documentation whether each test was prompted by patient-reported symptoms, including anal mass, pain, bleeding, itching, or discharge. We defined three groups: (1) a screened cohort, who underwent asymptomatic screening during the study period, (2) a never-screened, symptom-driven cohort, which included people who had anal cytology or HRA performed as a diagnostic procedure to evaluate their symptoms or exam findings, and (3) a never-tested cohort, who never underwent any anal cytology nor HRA tests. We analyzed all cytology and histology results from screening and diagnostic testing.

### Statistical Analysis

We compared characteristics between screened and unscreened groups using Wilcoxon rank-sum and chi-squared test or Fisher's exact tests. We estimated the prevalence of HSIL and anal cancer, and used univariable and multivariable mixed effects logistic regression to assess factors associated with abnormal cytology or histology. We conducted statistical analyses in RStudio (Version 2024.04.2) with *P* < .05 considered statistically significant.

## RESULTS

**Figure 1.**
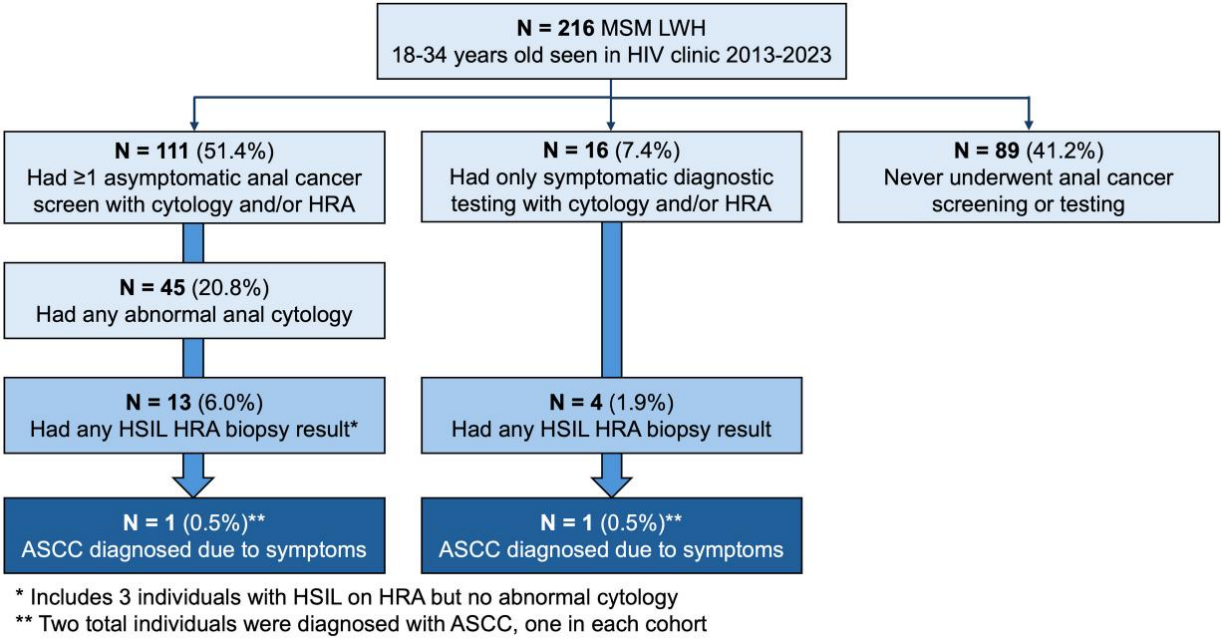
Individual-level outcomes by screening status for the population of men who have sex with men living with HIV aged 18–34.

A total of 216 MSM LWH had at least one clinic visit between ages 18 and 34, of whom 111 (51.4%) had at least one screening encounter (Figure 1). Table 1 describes the population characteristics. The median age was 26.7 years. Non-Hispanic White individuals comprised 24% of the cohort, 44% were Non-Hispanic Black, and 26% were Hispanic. Seventy-five percent had CD4 T-cell count ≥500 cells/mm^3%^ and 5% had CD4 T-cell count <200 cells/mm^3^. Eighty-eight percent had HIV viral load <1000 copies/mL at their last visit. Nearly half (46%) had received at least one dose of HPV vaccine. Median follow-up duration was 4.0 years (IQR 1.9–6.2 years) for the screened cohort and 1.6 years (IQR 0.7–4.2 years) for the never tested cohort. Sixteen individuals who were never screened did undergo anal cytology or HRA as symptom-driven diagnostic testing (Figure 1).

**Table 1.** Demographics and Characteristics of 216 Men Who Have Sex With Men Living With HIV Aged 18–34, Stratified by Anal Cancer Screening History During a 10-Year Period

Characteristic	Total, N = 216	Screened, N = 111	Diagnostic Testing Only, N = 16	Never Tested, N = 89	*P* Value^a^
Age at first visit, years	…	…	…	…	0.351
18–24	70 (32%)	40 (36%)	2 (13%)	28 (31%)	…
25–29	80 (37%)	39 (35%)	9 (56%)	32 (36%)	…
30–34	66 (31%)	32 (29%)	5 (31%)	29 (33%)	…
Race/Ethnicity	…	…	…	…	0.825
Non-Hispanic White	51 (24%)	28 (25%)	4 (25%)	19 (21%)	…
Non-Hispanic Black	94 (44%)	44 (40%)	7 (44%)	43 (48%)	…
Hispanic	56 (26%)	29 (26%)	5 (31%)	22 (25%)	…
Other	15 (6.9%)	10 (9.0%)	0 (0%)	5 (5.6%)	…
Total follow-up duration, years	3.12 (1.23, 5.16)	4.01 (1.92, 6.15)	3.64 (0.73, 4.10)	1.64 (0.74, 4.24)	<0.001
Insurance	…	…	…	…	0.321
Private	88 (41%)	50 (45%)	6 (38%)	32 (36%)	…
Public (Medicare or Medicaid)	104 (48%)	47 (42%)	10 (63%)	47 (53%)	…
None	24 (11%)	14 (13%)	0 (0%)	10 (11%)	…
Last CD4 T-cell count	…	…	…	…	0.281
>499	162 (75%)	89 (80%)	11 (69%)	62 (70%)	…
200–499	42 (20%)	18 (16%)	3 (19%)	21 (24%)	…
<200	11 (5.1%)	4 (3.6%)	2 (13%)	5 (5.7%)	…
Last HIV viral load	…	…	…	…	0.317
<20	142 (66%)	80 (72%)	9 (56%)	53 (60%)	…
20–1000	48 (22%)	20 (18%)	5 (31%)	23 (26%)	…
>1000	26 (12%)	11 (9.9%)	2 (13%)	13 (15%)	…
Had HPV vaccine	99 (46%)	53 (48%)	5 (31%)	41 (46%)	0.464
Age at first vaccine	25 (21, 30)	24 (21, 30)	28 (27, 30)	25 (20, 30)	0.482
Current or former smoker	105 (49%)	51 (46%)	8 (50%)	46 (52%)	0.717

Data are presented as number of cases (percent) or median (IQR). *P* value <0.05 is considered as statistically significant.

HPV, human papillomavirus.

^*a*^Pearson's χ^2^ test; Fisher's exact test.

### Descriptive Analysis of Test Results

Of 127 total tested individuals (111 asymptomatic and 16 symptom-prompted), the initial test result was 65 (51.2%) negative, 23 (18.1%) ASC-US, 18 (14.2%) LSIL, 2 (1.6%) HSIL, and 19 (15.0%) unsatisfactory. The initial screening result among the 111 individuals with an asymptomatic screen was 61 (55.0%) negative, 19 (17.1%) ASC-US, 12 (10.8%) LSIL, 1 (0.9%) HSIL, and 18 (16.2%) unsatisfactory. In total, there were 246 anal cytology tests performed (allowing for multiple tests per individual), of which 76 (30.9%) were abnormal: ASC-US (49; 19.9%), LSIL (24; 9.8%), and HSIL (3; 1.2%). Forty-six (46; 19%) were unsatisfactory. Sixty-seven individuals (67; 52.8%) underwent one cytology test, 27 (21.3%) underwent two, and 31 (24.4%) underwent three or more. From the 76 abnormal cytology tests, 34 (44.7%) led to HRA referral and 23 (30.3%) completed HRA. Cytology results preceding these 23 HRA procedures were 13 (56.5%) ASC-US, 8 (34.8%) LSIL, and 2 (8.7%) HSIL. The HRA biopsies revealed 6 (26.1%) negative, 6 (26.1%) with LSIL, 10 (43.5%) HSIL, and 1 (4.3%) HSIL with SCC foci (Table 2). The ASCC case was preceded not by screening, but rather a symptom-driven diagnostic testing encounter. Preceding cytology showed LSIL in the setting of anal bleeding and large condyloma. Among the 53 abnormal cytology tests which were not immediately followed by HRA, 28 (52.8%) were followed up with repeat cytology.

**Table 2.** Results of All Anal Cytology and of High Resolution Anoscopy Tests Following Abnormal Cytology Performed on Men Who Have Sex With Men Living With HIV Aged 18–34 in a 10-Year Period

	Anal Cytology (n = 246)*^a^*		HRA (n = 23)*^a^*	
Characteristic	Asymptomatic screen (n = 208)	Symptoms prompting test (n = 38)	Asymptomatic screen (n = 12)	Symptoms prompting test (n = 11)
Cytology result				
Negative	108 (52%)	16 (42%)	…	…
ASCUS	39 (19%)	10 (26%)	…	…
LSIL	15 (7.2%)	9 (24%)	…	…
HSIL	2 (1.0%)	1 (2.6%)	…	…
ASC-H	0 (0%)	0 (0%)	…	…
Unsatisfactory	44 (21%)	2 (5.3%)	…	…
HPV co-test result	…	…	…	…
Positive	39 (19%)	5 (13%)	…	…
Negative	23 (11%)	5 (13%)	…	…
Indeterminate	17 (8.2%)	2 (5.3%)	…	…
Not tested	129 (62%)	26 (68%)	…	…
Biopsy result	…	…	…	…
Negative	…	…	3 (25%)	3 (27%)
LSIL	…	…	3 (25%)	3 (27%)
HSIL	…	…	6 (50%)	4 (36%)
SCC*^b^*	…	…	0 (0%)	1 (9.1%)

Data are presented as number of cases (percent).

ASC-H, atypical squamous cells, cannot exclude high-grade squamous intraepithelial lesion; ASC-US, abnormal cells of uncertain significance; HPV, human papillomavirus; HRA, high resolution anoscopy; HSIL, high-grade squamous intraepithelial lesion; LSIL, low-grade squamous intraepithelial lesion.

^*a*^Some individuals had more than one test per person.

^*b*^A second incident SCCA case is not reported in this table due to diagnosis by direct HRA with no preceding cytology.

### Factors Associated With Abnormal Cytology/Histology

In mixed-effects logistic regression models (Table 3), tests prompted by symptoms (bleeding, itching, discharge) were associated with abnormal results (adjusted odds ratio [aOR] 8.54; 95% CI 2.55–28.67; *P* = .001). In the unadjusted model, individuals who received the HPV vaccine at or after age 26 were less likely to have abnormal results than those who were never vaccinated (OR 0.38; 95% CI 0.14–0.99; *P* = .048), though the association did not achieve significance in the multivariable model (aOR, 0.43; 95% CI 0.14–1.29; *P* = .13). Age was not associated with abnormal cytology or histology.

**Table 3.** Mixed Effects Logistic Regression Models for Factors Associated With Abnormal Cytology (ASC-US or Worse) or Abnormality on HRA Biopsy (LSIL or Worse) Among 18- to 34-Year-Old Men Who Have Sex With Men Living With HIV

		Univariable			Multivariable		
Characteristics	Abnormal Test, N = 76	Odds Ratio	95% CI	*P*-Value	Adjusted Odds Ratio	95% CI	*P*-Value
Age at test	…	…	…	…	…	…	…
18–24	19 (25%)	…	-	-	…	-	-
25–29	29 (38%)	1.35	0.55–3.32	0.51	1.39	0.47–4.12	0.55
30–34	28 (37%)	1.21	0.46–3.15	0.7	1.31	0.34–5.10	0.7
Race/ethnicity	…	…	…	…	…	…	…
Non-Hispanic White	22 (29%)	…	-	-	…	-	-
Non-Hispanic Black	25 (33%)	0.35	0.14–0.88	**0**.**03**	0.35	0.11–1.10	0.07
Hispanic	24 (32%)	0.74	0.26–2.08	0.57	1.09	0.31–3.82	0.89
Other	5 (6.6%)	0.45	0.09–2.21	0.32	0.77	0.13–4.44	0.77
Health insurance	…	…	…	…	…	…	…
Private	38 (50%)	…	-	-	…	-	-
Public	25 (33%)	0.57	0.25–1.32	0.19	0.52	0.19–1.42	0.2
None	13 (17%)	1.35	0.37–4.91	0.65	1.48	0.39–5.72	0.57
Years since HIV diagnosis	3.0 (1.0, 7.0)	0.91	0.81–1.02	0.1	0.91	0.80–1.04	0.18
Last CD4 T-cell count	…	…	…	…	…	…	…
≥500	63 (83%)	…	…	…	…	…	…
<500	13 (17%)	0.95	0.36–2.53	0.92	0.54	0.17–1.70	0.29
HIV viral load	…	…	…	…	…	…	…
<20	51 (67%)	…	-	-	…	-	-
20–1000	17 (22%)	1.39	0.50–3.89	0.53	2.54	0.79–8.17	0.12
>1000	8 (11%)	1.4	0.33–5.91	0.65	2.19	0.48–9.96	0.31
Age at HPV vaccine*^a^*	…	…	…	…	…	…	…
Never vaccinated	43 (57%)	…	-	-	…	-	-
<26	18 (24%)	0.39	0.15–1.01	0.05	0.98	0.30–3.16	0.97
≥26	15 (20%)	0.38	0.14–0.99	**0**.**048**	0.43	0.14–1.29	0.13
Ever smoker	28 (37%)	0.61	0.28–1.36	0.23	0.65	0.26–1.63	0.36
Symptoms prompting test	21 (28%)	7.51	2.51–22.45	**<0**.**001**	8.54	2.55–28.67	**0**.**001**

*P* < 0.05 is considered as statistically significant (in bold).

ASC-US, abnormal cells of uncertain significance; HPV, human papillomavirus; HRA, high resolution anoscopy; LSIL, low-grade squamous intraepithelial lesion.

^*a*^Age 26 was selected as a clinical threshold given CDC guidelines recommending routine HPV vaccination through age 26, with subsequent diminished expected benefit.

An EHR query for ASCC cases revealed two individuals diagnosed with ASCC during the study period. The first, as described above, followed an LSIL cytology result conducted due to symptom burden. The second was diagnosed after direct HRA without preceding cytology, also due to high symptom burden. One individual was diagnosed at age 30 and one at age 29, both had CD4 T-cell nadir <20 cells/mm^3^ and neither had prior HPV vaccination. The first had no history of smoking, was diagnosed with HIV 7 years prior to ASCC diagnosis, and did not achieve viral suppression at any time from HIV diagnosis to ASCC diagnosis due to poor adherence to antiretroviral therapy; the second was an active smoker, had the HIV diagnosis for 5 years and was virally suppressed for 4 years. Both diagnoses were local/early-stage cancer and established by HRA performed for resection of large symptomatic condyloma. The first individual had undergone asymptomatic screening 4 years prior with ASC-US cytology but no HRA follow-up, and the second had never been screened for ASCC. Of note, both individuals had a longstanding history of poor medication and follow-up adherence to HIV care, and neither completed the full course of recommended diagnostic testing or treatment with chemoradiation. A review of the Yale Tumor Registry did not reveal any additional ASCC cases in this population and study dates.

## DISCUSSION

In this retrospective cohort study, we evaluated the burden of precancerous anal disease and the uptake of anal cancer screening among 216 MSM LWH aged 18–34 over a 10-year period. Our findings highlight four major issues: (1) modest uptake of screening; (2) significant variability in and high rates of unsatisfactory cytology; (3) a high prevalence of histologic HSIL and the role HPV vaccination; and (4) the rarity of ASCC in this age group and importance of host-level risk factors in cancer progression (low CD4 nadir and low CD4:CD8 ratio, smoking and poorly controlled HIV viremia) as well as symptom-driven diagnostic testing.

Anal cytology performed poorly as a screening test in this population. Ideally, screening tests should be highly sensitive and can tolerate a relatively low specificity [18]. However, a 2022 meta-analysis reported that HSIL anal cytology had a remarkably limited sensitivity of only 24.6% for the detection of histological precancer among MSM LWH [19, 20]. To improve sensitivity, the cytologic threshold for follow-up can be lowered to include any abnormality (ie, ASC-US), now a standard protocol adopted by our clinic, and HPV co-testing added. In the meta-analysis, these changes increased sensitivity for HSIL detection to 85.2% and 93.0%, respectively, but at the cost of severely decreased specificity (52.8% and 33.4%, respectively) [19]. Additionally, evidence suggests that anal cytology consistently underestimates the degree of dysplasia seen on HRA, which was also demonstrated in our cohort [20–22]. There were only three cases of HSIL results by cytology, but 11 (47.8%) HSIL or SCC results following an abnormal cytology result. Furthermore, the high rate of unsatisfactory cytology results (19%) in our cohort highlights the technical challenges of this test. This is consistent with the 17% unsatisfactory cytology rate observed by Liu et al. in a prior study of a similar population aged <35 and reports in older cohorts of 10–20% [6, 20, 23, 24]. Unsatisfactory results were even more common among asymptomatic screening encounters (21%) compared to tests prompted by symptoms (5.3%), which were more likely to yield abnormal results. The unsatisfactory rate is an order of magnitude higher than that seen with cervical cytology, to which anal cytology is often compared [25, 26]. It is unclear to what extent this discrepancy arises from intrinsic test limitations, provider inexperience, or host factors and anatomical challenges.

Importantly, access to HRA is highly limited, even in resource-rich settings, necessitating a screening test with good discriminatory power to effectively triage individuals requiring further evaluation [27, 28]. A proportion of abnormal cytology results were not followed by HRA in our cohort, which reflects the challenges in adherence and real-world implementation of routine HRA use. It likely also reflects practice variation before the new guidelines (eg not consistently referring low risk or ASC-US results and rather following by repeat cytology). Also, some individuals may have undergone HRA after age 34 which would not be included in our analysis. Some individuals were referred to colorectal surgery for HRA but did not make or attend the appointment, while others were never referred. This may have been due to patient preferences and shared clinical decision-making. Adherence to HRA referrals may also be limited by documented challenges with longitudinal engagement in HIV care [29]. Unfortunately our study was limited by lack of individual-level data on why some individuals could not get timely HRA.

The prevalence of histologic HSIL in our cohort (43% among those undergoing HRA) is consistent with a previous study of 1255 MSM LWH aged 34 and younger, which reported HSIL prevalence of 47% [6]. The prevalence of HSIL among young MSM LWH overall is expected to be lower than that in this prescreened population with prior abnormal cytology. However, even among a nonenriched population of young MSM LWH, HSIL rates are as high as 34% [30]. These findings reaffirm the high burden of HPV-associated disease in this population. Of note, while cytologic abnormalities and histologic HSIL are common in younger MSM LWH, some evidence suggests that spontaneous HSIL clearance may occur more frequently in younger individuals [6, 31–33]. Persistent infection with HPV, particularly HPV-16, is a well-established risk factor for anal cancer, and HPV infection rates approach 90% among MSM LWH [34, 35]. However, because the majority of screening encounters in our cohort (63%) did not co-test for HPV, we did not interpret HPV prevalence in this study.

Vaccination uptake in our cohort was modest. Less than half (46%) of our population had received at least one dose of the HPV vaccine, which is higher than US population-based estimates of MSM at 13–37%, but still lags behind national averages for overall vaccination rates [36–38]. Although HPV vaccination was associated with a trend toward reduced abnormal test rates in our unadjusted analyses, this effect did not persist after adjustment, though our study is limited by small sample size. The high prevalence of prior HPV exposure among MSM LWH likely diminishes vaccine efficacy in this population [39]. Nevertheless, there are seven oncogenic types covered in the nonavalent HPV vaccine, and vaccination would protect against the remaining types if a person has not been infected with all seven. Increasing vaccination rates through organized public health efforts remains an imperative goal to preventing HPV-associated disease in MSM LWH.

While histologic HSIL was frequently identified following abnormal cytology, we identified only two cases of early-stage local ASCC in our cohort, neither of which were identified by screening pathways. Rather, both individuals presented with clinical symptoms and large condyloma prompting diagnostic evaluation. A study by Liu et al reported no cases of anal cancer among more than 1200 MSM LWH under age 35 undergoing asymptomatic screening [6]. In our cohort, screening encounters likewise did not detect any ASCC cases. While the occurrence of any cancer cases in this young cohort is noteworthy, the cases in our cohort fall in a high-risk group separate from asymptomatic screening given their high symptom burden and severe immunosuppression (CD4 T-cell nadir <20 cells/mm^3^). Overall, we suggest a low incidence of progression from precancerous to cancerous lesions among this young age group, consistent with prior studies [6]. An Australian natural history study found anal HSIL clearance of 22.0 per 100 person-years among MSM aged 35 and older, with higher clearance (29.8 per 100 person-years) associated with younger age [33]. Future studies should investigate which HSIL lesions are more likely to progress. While the ANCHOR study demonstrated that treating HSILs reduces the incidence of ASCC in MSM LWH aged 35 and older, evidence supporting similar benefits in younger individuals is limited [5]. In our study, asymptomatic screening in those aged 18–34 did not yield measurable benefits in terms of cancer prevention.

The presence of symptoms prompting testing was strongly associated with abnormal test results. This, along with the characteristics of the individuals diagnosed with ASCC, aligns with the established understanding that immunosuppression is a salient risk factor for anal cancer progression and suggests that risk stratification based on clinical symptomatology and immune status may be more effective than routine cytology-based screening in this demographic [40].

This study has several limitations. As a study limited to two urban academic clinics, our findings may not be generalizable to other populations. The retrospective design also introduces potential selection bias and relies on EHR data, which may not capture all vaccination encounters or screening records. Importantly, we could not analyze detailed individual-level reasons on why some individuals did not get initial screening and why some abnormal tests were not followed by HRA from the chart. Additionally, the relatively small number of HRA procedures and ASCC cases limits our ability to draw definitive conclusions about the association between screening practices and cancer outcomes. Furthermore, the high rate of unsatisfactory cytology and low follow-up rate after abnormal results could have led to underdiagnosis. Because only a subset of individuals underwent cytology and HRA, our estimates represent test positivity among those evaluated, which may not reflect true population-level prevalence. To counter this, we did evaluate the broader Tumor Registry, which should capture all cases diagnosed in our system.

In conclusion, while anal cancer screening in MSM LWH aged 18–34 identifies a high burden of precancerous disease, the low progression rate to invasive cancer and significant limitations of current screening tools and limited resources for HRA, all support an age-based cutoff for routine asymptomatic anal cancer screening. Expanding routine anal cytology-based screening may result in overdiagnosis, unnecessary procedures, and increased healthcare costs without clear benefits in cancer prevention. Clinicians should continue to inquire about and evaluate anal symptoms. Evaluation of symptomatic individuals, especially in higher risk groups, may proceed directly to HRA, forgoing cytology, to improve diagnostic yield and resource efficiency. Further research, including randomized clinical trials powered for clinical outcomes, is needed to better understand the natural history of HSILs in young individuals and to optimize strategies to reduce SCCA morbidity without undue burden on patients and the healthcare system.
